# Low breastfeeding rates and body mass index in Danish children of women with gestational diabetes mellitus

**DOI:** 10.1186/s13006-015-0051-8

**Published:** 2015-09-09

**Authors:** Jesper Fenger-Grøn, Morten Fenger-Grøn, Charlotte Holst Blunck, Helena Schønemann-Rigel, Hanne Benedicte Wielandt

**Affiliations:** Department of Paediatrics, Lillebaelt Hospital, 6000 Kolding, Denmark; Research Unit for General Practice, Aarhus University, 8000 Aarhus C, Denmark; Department of Gynaecology and Obstetrics, Lillebaelt Hospital, 6000 Kolding, Denmark; Institute of Regional Health Service Research, University of Southern Denmark, 5000 Odense, Denmark

## Abstract

**Background:**

Offspring from women with gestational diabetes mellitus (GDM) are at risk for later overweight, and the aim of treatment regimens is to normalize their prognosis. While the general concept is that breastfeeding is protective and should be promoted, some studies report increased levels of insulin and glucose in breast milk of women with diabetes, possibly increasing risks to the children. Previous studies may have low retention rates or mix GDM and pre-GDM, and often knowledge of confounders like maternal body mass index (BMI), level of hyperglycemia and feeding patterns is lacking. Data on breastfeeding rates, growth patterns and their associations are important to optimize future strategies among offspring from women with GDM managed by diet.

**Methods:**

Based on 10.730 births, a cohort of 131 singletons of Danish women with GDM managed by diet was defined. Data on feeding patterns, offspring length, weight and head circumference were obtained at the initial admission and from examinations by the general practitioner at five weeks and at five months postpartum. Breastfeeding rates were described in relation to neonatal and maternal characteristics and compared to national rates, while anthropometric data were compared to reference standards. The association between breastfeeding and offspring growth was analysed with and without correcting for confounding.

**Results:**

More than 99 % of the cohort contributed to anthropometric data, while data on feeding patterns were available for 96–98 %. Of mothers, 8 % did not initiate breastfeeding and the rate of fully breastfeeding at five weeks and at five months of age were 61 % and 18 %, respectively, which is considerably lower than generally reported in Denmark. Lowest breastfeeding rates were seen following prelabour Caesarean delivery. Complementary feeding was introduced earlier than recommended among 11 %. At the age of five weeks and at five months, children had grown longer and had lower BMI than expected from Danish and World Health Organization references. In the study periods, breastfeeding was significantly associated with lower BMI.

**Conclusion:**

Despite lower breastfeeding rates than normally reported in Denmark, offspring BMI at the age of five months were low. Still new initiatives to promote breastfeeding among Danish women with GDM should be considered.

## Background

Diabetes during pregnancy is increasingly becoming a major health problem, affecting a large and continuously increasing number of women [1, 2], and the vast majority of cases are gestational diabetes mellitus (GDM) defined as glucose intolerance with onset or first recognition during pregnancy [2, 3]. Obesity increases the risk of a pregnancy complicated with GDM, but GDM may also be diagnosed among non-obese pregnant women. Intrauterine exposure to maternal hyperglycemia seems to place offspring at increased risk for long-term adverse outcomes like overweight and diabetes [1, 2, 4, 5], and likewise maternal obesity may place the offspring at similar risk [2, 6]. Among children of women with GDM special attention has been given to the growth pattern during the first months of life, as increased early weight gain has been reported as a strong, independent risk factor for childhood overweight [7].

Breastfeeding is the natural and best way to nurture healthy offspring of healthy mothers [8–10], and observational studies suggest a lower risk of later overweight, hypercholesterolemia, hypertension and type 2 diabetes among breastfed than among formula fed newborns [8, 11]. However, breast milk from women with diabetes mellitus has been shown to have higher insulin and glucose levels and a cohort study regarding offspring from women with diabetes mellitus during pregnancy found an association between early ingestion of mother’s breast milk and overweight later in childhood [1]. These studies combine GDM and pre-GDM, and other studies are criticized for lack of knowledge of important confounders like maternal pregestational body mass index (BMI) or the level of intrauterine hyperglycemic exposure [2]. Selection bias is also an imminent problem, some studies include only motivated families [1], other studies being significantly weakened by the loss to follow-up around 50 % or more [12–14]. International literature lacks observational studies on children of women with GDM where the retention rate is high, the pregnancy including the level of intrauterine hyperglycemic exposure is well-described, and feeding patterns, maternal BMI and lifestyle are known, and such studies have been requested [2]. In Denmark deliveries are normally on an out-patient basis, though GDM patients are offered hospital admission for some days following delivery to ensure counselling in breastfeeding and maximizing the chance of the successful initiation of breastfeeding. The overall intention of treatment is to transform the chances of offspring from a group characterized by high risks of neonatal adverse outcomes, breastfeeding difficulties and later overweight, into a group similar to the background population. However, even though considerable resources are allocated to treatment of GDM patients during pregnancy and the above mentioned breastfeeding promoting regimens, neither the impact on breastfeeding rates nor infant growth patterns have been thoroughly evaluated, and currently it is unknown if breastfeeding rates and growth patterns still differ significantly from the background population.

The aim of the present study was to follow-up a regional cohort of offspring from a homogenous group of nulliparous women with GDM managed by lifestyle interventions and to analyse whether growth, feeding patterns and associations during the first five months of life differed from the background population.

## Methods

### Study sample

The present cohort study concerned singleton offspring from nulliparous women diagnosed with gestational diabetes and managed by lifestyle interventions without insulin, delivered at The Department of Gynecology and Obstetrics, Lillebaelt Hospital, Kolding during the period from 1 January 2010 until 30 April 2013. The only exclusion criterion was a language barrier.

In the study period, 132 potentially eligible singletons were delivered, but a single child was excluded as the mother was neither Danish nor English speaking. The remaining 131 children were included in the following analysis of growth and feeding patterns. The background population consisted of 10.730 children born at Lillebaelt Hospital, Kolding in the period.

### Screening for GDM and antenatal care

In accordance with the Danish routine screening procedure for GDM [15], nulliparous pregnant women with pre-pregnancy BMI > 27 kg/m^2^ (BMI calculated as weight in kilos divided by height^2^ in meters^2^), family history of diabetes, or polyhydramnios were invited to have a 75 g oral glucose tolerance test, and GDM was diagnosed if the two hour blood glucose level was above 9.0 mmol/L.

The antenatal care is described in detail elsewhere [15, 16], in brief: The women with GDM were offered an initial consultation with an obstetrician giving information on GDM, clinical examination including measurement of blood pressure, urine-analysis and an ultrasonographic measurement in order to follow foetal growth. The GDM patients returned for follow-up visits with intervals of two-six weeks throughout their pregnancies. Depending on the circumstances, the health care professional at the consultation could be a specialized diabetic nurse, an obstetrician, or a dietician. Supervision by an endocrinologist was always possible. At one of the initial obstetrical visits the GDM patients were informed of the study, and they all gave consent to participate in the study.

The goal for treatment was a self-monitored blood glucose level between 4–6 mmol/L pre-prandially and 4–8 mmol/L post-prandially and an HbA1C < 5.6 % [15]. Insulin treatment was initiated if the GDM patient presented blood glucose values exceeding the treatment goal. These patients were referred to The Regional Centre for Pregnant Women with Diabetes Mellitus, University Hospital of Odense.

### Breastfeeding counselling and postnatal care

The Danish National Board of Health recommends and assumes that breastfeeding counselling is an integrated part of consultations during all pregnancies [9], but no specific guideline is made. Following labour, the women with GDM and their offspring are offered hospital admission for a few days. The purpose is to avoid offspring hypoglycemia and to help establish breastfeeding.

### Data on feeding patterns and anthropometrics

In Denmark newborns are offered free consultations by the family’s general practitioner at five weeks and again at five months after delivery, and in general the retention rate is very high. At these times, length, weight and head circumference are measured and registered along with feeding patterns. In Denmark birth data are reported to The National Board of Health, but in contrast, data from the primary health service can only be collected through direct contact to families or general practitioners. At birth, newborns get a medical record which should accompany the child whenever the health care system is involved during childhood, and this book comprises copies of the general practitioner’s record as well as comments from the health care visitor.

Between 1st of September, 2013 and 1st of November, 2013 mothers of all included children were contacted on the telephone number registered at the obstetrical consultation. At this time the children were between six months and 3½ years old. In case of initial non-response, several phone calls were made at different times during the next week and supplemented by contacts via Short Message Service (SMS) or by letter, requesting a call-back to the research team. At the telephone interview, the mother was asked to report data on length, weight, head circumference and feeding patterns registered in the medical record at the examinations by the general practitioner. If necessary, these data were collected directly from the general practitioner. In a few cases with missed registration of feeding patterns at the examination, information registered from the health care visitor or deriving from the maternal interview was accepted. Fully breastfeeding was defined according to The National Board of Health [9] to allow supplement by water or formula no more than once a week. Feeding information was collected on breastfeeding initiation, feeding patterns at the time of examination by the general practitioner (fully breastfeeding/partly breastfeeding/no breastfeeding), total time of breastfeeding (any) and timing of the introduction of complementary feeding like porridge or cooked, mashed vegetables or fruit.

Data on maternal smoking habits and pregestational BMI potentially confounding the association between feeding patterns and growth were obtained from the general practitioner’s referral to the hospital, while data concerning delivery and the newborn child were obtained from the hospital record.

### Ethical considerations

The women with GDM were informed of the study at one of the initial obstetrical visits, and they all gave verbal consent of contribution. According to the head of The Research Ethics Committee of The Southern Denmark Region, the Danish Act on Research Ethics Review of Health Research Projects did not apply to this study, and consequently the study did not need further approval from the committee.

### Analysis

**Fig. 1:**
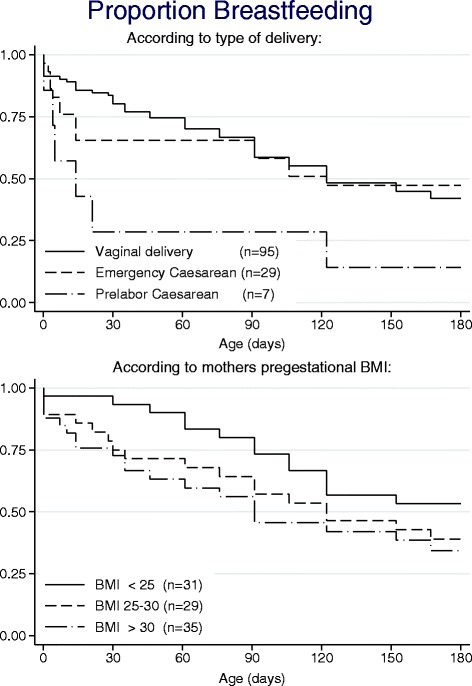
Proportion breastfeeding according to delivery mode and pregestational BMI. Kaplan-Meier curves of breastfeeding related to delivery mode and maternal pregestational BMI. Prelabour Caesarean delivery versus vaginal delivery *p =* 0.01 (log-rank test). Among vaginal deliveries: Maternal BMI and breastfeeding *p =* 0.067 (Cox regression)

Curves presenting the proportion of breastfed children as a function of age (Fig. 1) were drawn using the Kaplan-Meier approach including the standard assumption of non-informative censoring and subgroups were compared by log-rank test. Testing for linear trend between ordered groups (maternal pregestational BMI) was carried out applying a Cox regression model. Comprehensive data on breastfeeding rates in relation to important maternal and neonatal characteristics are presented in Table 1.

**Table 1:** Breastfeeding status at five weeks and at five months of age

5 weeks of age		Fully breastfed (*n =* 78)	Partly breastfed (*n =* 16)	Not breastfed at all (*n =* 34)	Unknown (*n =* 3)
Sex	Boys	42	6	22	2
	Girls	36	10	12	1
Maturity	Premature	5	0	1	0
	Term	73	16	33	3
Neonatal hypoglycemia	Yes	11	0	8	0
	No	67	16	26	3
Birth weight	SGA	3	1	2	0
	AGA	74	14	28	3
	LGA	1	1	4	0
Delivery mode	Vaginal	59	14	19	3
	Prelabour Cs	2	0	5	0
	Emergency Cs	17	2	10	0
Maternal smoking	Yes	4	2	8	0
	No	74	14	26	3
Maternal BMI	<25	31	6	5	1
	25–30	21	5	10	1
	≥30	26	5	19	1
5 months of age		Fully breastfed (*n =* 23)	Partly breastfed (*n =* 34)	Not breastfed at all (*n =* 68)	Unknown (*n =* 6)
Sex	Boys	11	16	41	4
	Girls	12	18	27	2
Maturity	Premature	1	2	3	0
	Term	22	32	65	6
Neonatal hypoglycemia	Yes	3	3	13	0
	No	20	31	55	6
Birth weight	SGA	0	1	5	0
	AGA	22	33	58	6
	LGA	1	0	5	0
Delivery mode	Vaginal	14	29	47	5
	Prelabour Cs	1	0	6	0
	Emergency Cs	8	5	15	1
Maternal smoking	Yes	0	2	12	0
	No	23	32	56	6
Maternal BMI	<25	11	13	18	1
	25–30	4	14	18	1
	>30.0	8	7	32	4

Abbreviations: *Cs* Caesarean section, *SGA* small for gestational age, *AGA* appropriate for gestational age, *LGA* large for gestational age, *BMI* body mass index (pregestational)

**Fig. 2:**
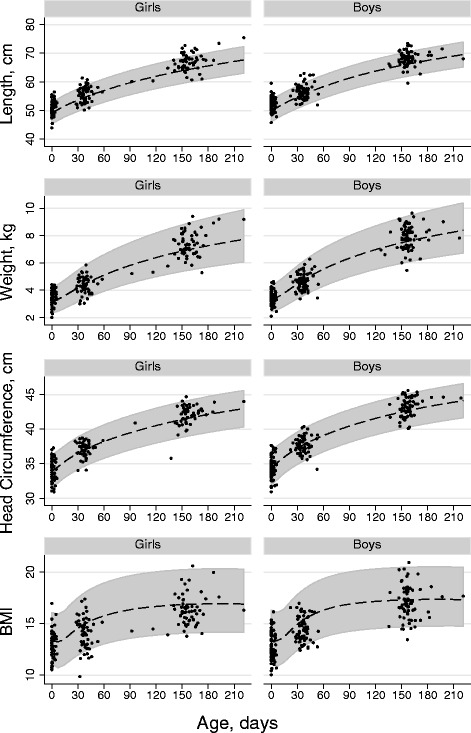
Growth of offspring from women with GDM. Anthropometric data in a cohort of offspring from women with GDM at birth, at about five weeks and at five months of age superimposed on WHO’s childhood reference material. Girls and boys developed significantly increased length and significantly lower BMI than expected (statistics given in Table 2)

Table 2 presents the statistical analyses of anthropometric measurements. The analyses were carried out applying a normal distribution model on the standardized scores (SD-scores or z-scores) calculated using a Box-Cox transformation [17] where the LMS parameters are the median (M), the generalized coefficient of variation (S), and the power in the Box-Cox transformation (L) for the reference populations [18, 19]. Statistical analyses were carried out based on both WHO growth standards [18] and the newly published Danish references [19] as it is currently unclear which should be used for scientific purposes during the first months of life in a Danish setting [9, 19, 20]. It was only considered clinically relevant if anthropometric measures were consistently significant. For the WHO standards, age-specific parameters were given for each day [18], while for the Danish reference levels linear interpolation to each day was made based on parameters given in steps of 1/100 years [19]. Thus, the scale unit for the estimates is one standard deviation for the scores that are assumed standard normally distributed in the reference material. The WHO growth standards were chosen as background for Fig. 2, illustrating the anthropometric measures.

**Table 2:** Statistics on length, weight, head circumference and BMI at follow-up of offspring from women with GDM

			Compared to WHO reference				Compared to Danish reference			
			Estimate	95%-CI		p-value	Estimate	95%-CI		p-value
At age appr. 5 weeks	Length									
		Boys	0.61	0.33	0.89	<0.001***	0.19	-0.06	0.44	0.14
		Girls	0.68	0.30	1.05	0.001**	0.37	0.02	0.72	0.04*
	Weight									
		Boys	-0.17	-0.39	0.55	0.14	-0.26	-0.48	-0.05	0.02*
		Girls	-0.11	-0.40	0.17	0.42	-0.19	-0.48	0.11	0.21
	Head circ.									
		Boys	0.07	-0.18	0.31	0.60	-0.37	-0.58	-0.16	<0.001***
		Girls	0.12	-0.15	0.40	0.36	-0.15	-0.40	0.10	0.23
	BMI									
		Boys	-0.71	-0.94	-0.50	<0.001***	-0.62	-0.83	-0.41	<0.001***
At age appr. 5 months		Girls	-0.66	-0.96	-0.36	<0.001***	-0.65	-0.99	-0.30	<0.001***
	Length									
		Boys	0.86	0.60	1.13	<0.001***	0.52	0.27	0.76	<0.001***
		Girls	1.17	0.87	1.47	<0.001***	0.79	0.50	1.08	<0.001***
	Weight									
		Boys	0.29	0.06	0.52	0.01*	-0.01	-0.22	0.20	0.93
		Girls	0.33	0.09	0.59	0.008**	0.04	-0.02	0.29	0.72
	Head circ.					p-value				
		Boys	0.41	0.16	0.67	0.002**	-0.89	-0.32	0.14	0.44
		Girls	0.42	0.12	0.72	0.007**	-0.04	-0.35	0.26	0.78
	BMI									
		Boys	-0.27	-0.53	0.0	0.046*	-0.36	-0.61	-0.11	0.005**
		Girls	-0.41	-0.66	-0.16	0.002**	-0.53	-0.79	-0.27	<0.001***

Abbreviations: *GDM* Gestational diabetes mellitus, *BMI* body mass index, *95%-CI* 95 % confidence interval. Estimates are given on the standardized scale (SD-score). *p < 0.05, **p < 0.01, ***p < 0.001

In the multivariable analysis of breastfeeding rates and anthropometric measurements (Table 3), children receiving breastfeeding for only part of the period were treated as 50 % exposed to breastfeeding. In supplementary analyses this linearity assumption was substituted by a model considering them as an independent level of a categorical variable. For the period five weeks to five months a subcategorization of the group not breastfed at five months according to their status at five weeks was considered as well.

**Table 3:** Associations between breastfeeding and anthropometric data in a cohort of offspring from women with GDM

		Crude				Adjusted			
		Estimate	95%-CI		p-value	Estimate	95%-Cl		p-value
Period 0–5 weeks:									
	Weight	−0.28	−0.54	−0.03	0.027*	−0.23	−0.51	0.04	0.090
	Length	0.25	−0.14	0.64	0.206	0.26	−0.15	0.68	0.210
	BMI	−0.59	−0.95	−0.22	0.002**	−0.54	−0.93	−0.15	0.007**
	Head circumference	−0.34	−0.71	0.03	0.075	−0.36	−0.76	0.04	0.075
Period 5 weeks to 5 months:									
	Weight	−0.60	−0.93	−0.26	0.001**	−0.57	−0.92	−0.23	0.001**
	Length	−0.31	−0.73	0.11	0.143	−0.27	−0.71	0.17	0.223
	BMI	−0.60	−1.02	−0.18	0.006**	−0.59	−1.04	−0.14	0.011*
	Head circumference	−0.16	−0.58	0.25	0.436	−0.08	−0.51	0.36	0.721

Associations between breastfeeding and anthropometric measures in the study periods from birth to five weeks of age and five weeks to five months of age. Estimates are given on the standardized scale (SD-score), and adjusted values are corrected for maternal pregestational BMI and smoking status. All values are adjusted for the child’s SD-score at beginning of each period. *p < 0.05, **p < 0.01. For further interpretation see text

Mothers were divided into three groups according to pregestational BMI (BMI < 25, 25 ≤ BMI < 30, BMI ≥ 30) and associations between maternal BMI and offspring BMI at five months of age were tested using pairwise comparisons.

P-values are two-sided, and in general p-values less than 0.05 were considered statistically significant. Analyses were carried out using the statistical software package Stata 12.1, StataCorp LP, USA.

## Results

Maternal and neonatal background data are described in details elsewhere [21] and can be extracted from Table 1.

At the age of five weeks, seven newborns did not consult the general practitioner, and nine head circumferences were not recorded. However, data on feeding patterns could be supplied by maternal information making feeding patterns at the age of five weeks available for 128/131 children.

At the age of five months, 129 of 131 children were examined by the general practitioner, and data on child number 130 could be obtained from admission to the local pediatric department because of infection at the age of four months and two weeks. The last child had emigrated shortly after birth and could not be traced in spite of phone calls, SMS and letters. All 130 children had their weight and length measured, while data on feeding patterns were unavailable in relation to five of the children, and head circumference was not recorded in relation to 15 consultations. All together length and weight at the age of about five months were available for 130/131 (99 %) of the children of the women with GDM and feeding patterns were available for 125/131.

### Feeding patterns

Table 1 presents breastfeeding data from the time of the examination by the general practitioner at age five weeks and at five months. Breastfeeding is divided into “fully”, “partly” or “no” and is presented in relation to sex, maturity, neonatal hypoglycemia, birth weight, delivery mode, maternal smoking and maternal BMI. At the age of five weeks 78/128 (61 %) of the cohort were fully breastfed, 16/128 (12 %) were partly breastfed while 34/128 (27 %) were not breastfed at all.

At the age of five months 23/125 (18 %) were fully breastfed, 34/125 (27 %) were partly breastfed while 68/125 (54 %) of mothers had totally stopped breastfeeding. Data on introduction of complementary feeding could be obtained regarding 118/131 of the families, and porridge or mash was in general introduced at about the age of five months, however 13/118 (11 %) reported introduction prior to the age of four months. Never having initiated breastfeeding at all was reported by 10/128 (8 %) of mothers.

Data presented in Table 1 indicated an association between neonatal hypoglycemia and breastfeeding difficulty, see discussion for further interpretation. The revealed association between breastfeeding rates and mode of delivery or maternal pregestational BMI was further analyzed, Fig. 1 shows Kaplan-Meier curves. Here all sorts of breastfeeding (fully as well as partly) are considered together. In spite of the small numbers, a significant difference between breastfeeding rates following vaginal and prelabour Caesarean delivery could be disclosed. Likewise, a tendency for lower breastfeeding rates with increasing maternal pregestational BMI was seen.

### Anthropometric data

Figure 2 shows weight, length, head circumference and BMI superimposed on the published World Health Organization childhood growth curves [18]. Statistics on the anthropometric measurements are presented in Table 2 and as the WHO and Danish reference standards are different, the assessment of results obviously depended on choice of reference. Notably, at the age of five months the study population displayed significantly increased weight and head circumference compared with WHO standards, while compared with Danish standards the same measures were average or low. However at the age of five weeks and at five months the children had consistently grown longer and had a lower BMI compared with both reference populations.

At five months of age BMI did not differ significantly when comparing offspring from mothers with pregestational BMI below 25, between 25 and 30, and above 30, respectively (detailed analyses not shown).

### Breastfeeding and anthropometric data

Table 3 shows analyses of breastfeeding impact on measured anthropometric data. Analyses are presented as crude values as well as adjusted for maternal pregestational BMI and smoking status, but no clinically relevant differences could be displayed between the two. In the adjusted analysis, breastfeeding was significantly associated with a reduction in BMI SD score of 0.54 (approximately equivalent with 0.7 kg/m^2^) in the period from birth until five weeks of age, and a reduction in BMI SD score of 0.59 (approximately equivalent with 0.9 kg/m^2^) in the period from five weeks to five months of age. The downward shift in BMI trajectory was primarily explained by lower weight gain. Supplementary analyses showed no significant deviation from the linearity assumption of the partly breastfed groups being 50 % exposed, and only minimal changes of the results could be introduced by sub-categorizing the group not breastfed at five months according to their status at five weeks.

## Discussion

### Breastfeeding and feeding patterns

The breastfeeding rates reported in the present study were higher than among GDM patients in The United States [22] but were far from The Danish National Board of Health and WHO/UNICEF recommendations advocating full breastfeeding for the first six months of life [9, 10]. The observed rates were also considerably lower than normally reported in Denmark, the most quoted Danish survey [23] found less than 5 % of offspring not being breastfed at discharge, and the rate of fully breastfeeding at five weeks and at five months of age being 77 % and 32 %, respectively, corresponding to 8 %, 61 % and 18 % in our cohort. Recently, the median time of fully breastfeeding in Danish offspring was reported to be 122 days [24], while in our cohort the rate only had a comparable magnitude if fully and partly breastfeeding were considered together (Fig. 1). Complementary feeding was introduced early, 11 % even prior to the earliest official recommendation at the age of four months [9].

Previously, breastfeeding rates at hospital discharge have been shown to be lower after Caesarean than vaginal birth [23, 25], and recently it has been suggested that Caesarean birth prior to labour should be associated with the highest rates of breastfeeding difficulties [25]. Present data supports this observation (Fig. 1). A labour-related endocrine process to initiation of milk production has been postulated as a biological plausible explanation for the breastfeeding difference [25], and counselling of mothers requesting Caesarean delivery without medical indication should include information on potential adverse consequences of Caesarean birth beyond the intrapartum period [26].

In general breastfeeding difficulties are well-known among women with GDM [27] and overweight mothers [9, 28], and nulliparous Danish women have been reported to have marginally lower breastfeeding rates than multiparous [23]. Consequently, some degree of breastfeeding difficulty could be anticipated when the Danish surveys are compared and this is the reason for offering postpartum admission including extra breastfeeding counselling as mentioned in recommendations from The Danish National Board of Health [9]. A specific procedure special to the children of the women with GDM is that they were all offered hypoallergic formula and a blood glucose test during the first hours following labour in order to avoid hypoglycemia, and Table 1 suggests an inappropriate effect from the early feeding regimen on breastfeeding initiation. Subsequently, the Danish national guideline on avoiding neonatal hypoglycemia has been fundamentally changed, but studies on the impact of the new guideline are still awaited.

The present group of women with GDM had received far more breastfeeding counselling than normal, and in spite of potential breastfeeding difficulties among this group of patients, we still consider lower breastfeeding rates compared with general Danish rates disappointing. This finding has not been reported previously.

### Anthropometric data

As anticipated [29], assessment of anthropometric data was sensitive to choice of WHO or Danish reference standards, and it is debated which should be used in a Danish setting [9, 19, 20]. We analysed anthropometric data on the basis of both reference populations and mainly considered measurements clinically relevant if results were consistently significant. The most remarkable study finding was the significantly increased length and low BMI at the age of five weeks and at five months.

Larger studies have demonstrated prenatal exposure to hyperglycemia combined with maternal overweight conveying an increased risk of offspring overweight [13, 30], and we stratified offspring into groups exposed to maternal GDM and different degrees of overweight to investigate if this was the case in our smaller study. Differences between groups were insignificant but showed the same tendency as reported elsewhere.

Increased weight gain during the first four months of life has been reported as a strong, independent risk factor for childhood overweight in offspring from GDM patients, and preventing nutritionally-induced rapid early weight gain has been mentioned as a promising strategy to lower their long-term overweight risk [7]. The mothers of the presented offspring cohort were in general well-regulated, and the low offspring BMI during the first five months of life is reassuring, but whether data should be considered the result of a successful intervention directed to the maternal life style during pregnancy, or whether it merely is affected by postnatal factors is unknown. The literature does not provide convincing evidence about a correlation between treatment of GDM and an improved offspring prognosis, but animal studies and human data strongly indicate that this could be the case [5]. Further follow-up of the cohort during childhood will provide additional evidence concerning the obesity risk in the cohort. Although associations of maternal impaired glucose tolerance and increased offspring size at birth is well-known [31], we are not aware of other publications reporting isolated significantly increased length during the first months of living.

### Anthropometric data and feeding patterns

There is an undisputed need to identify early determinants of obesity, and the association between breastfeeding and reduced risk of overweight and metabolic disturbances has been described as an outstanding example of the paradigm and concept of “perinatal programming” of health and disease [8]. Offspring from GDM patients also seem to benefit from breastfeeding [32], and interestingly evidence is being gathered that breastfeeding may even have positive effects on maternal prognosis [33, 34], further emphasizing the importance of high breastfeeding rates among GDM patients. Low breastfeeding rates combined with early introduction of complementary feeding among the study cohort of offspring from women with GDM indicate an increased risk of later overweight [15], and the present study confirms an association of breastfeeding and low weight gain, but the overall study finding of low BMI at the age of five months, regardless of breastfeeding status, is surprising. However, the perspectives are complex and for instance the only large-scale randomized trial on the effect of breastfeeding has challenged the predominant conception of breastfeeding leading to a downward weight and length trajectory [35]. Besides, a positive correlation between the neonatal intake of breast milk from diabetic mothers and later body weight has been reported [1], and increased concentrations of glucose and insulin, as well as a higher energy content of breast milk from women with diabetes, have been observed as compared with healthy mothers [1, 8, 36]. Potentially this might offer a dilemma to women with diabetes and their babies [1], and more research is needed to clarify whether breastfeeding might even have negative consequences with the risk of overweight and diabetogenic disturbances in the offspring of women suffering from glucose intolerance during pregnancy and lactation [8]. However, in the present study, the women with GDM had been vigorously well-regulated approaching metabolic status of healthy pregnant women, and in clinical practice the alternative to breastfeeding is not banked human milk but formula. Considering the variety of advantages resulting from breastfeeding in general, breastfeeding should remain the preferred type of infant feeding in offspring from diabetic patients [1].

### Retention rates

Data concerning the first five months’ of living from 99 % cohort of offspring from women with GDM is presented, and to our knowledge such a retention rate has not previously been reported in any other related study. However, there seems to be a dilemma between study particulars, cohort size and follow-up rate. Other well-conducted and larger follow-up studies on breastfeeding rates provided more detailed follow-up data but at the expense of the follow-up rate [1, 13, 25]. Studies reporting high retention rates faced selection bias including only the motivated families [37–39] at the study entry point. The impact of missing data on study conclusions is widely unknown, but it is reasonable to expect differences between families delivering follow-up data and non-responders. In the present study our priority has been to ensure data on the entire cohort, but a side effect was necessary restriction to available data on growth and feeding patterns from the examination by the general practitioner.

### Study limitations

The mothers of the present cohort had GDM and were successfully managed on diet and other lifestyle interventions, making the intrauterine environment metabolically well-described. It is not known if results can be generalized to offspring exposed to more extreme intrauterine hyperglycemia or insulin treatment.

The reliability of measurements in the Danish primary health care system is difficult to assess. Correct measurements of head circumference and weight are simple, and regarding length, many general practitioners have infantometers or measuring boxes and it is mandatory that all length measurements should be done recumbent by a trained health care professional. There is no reason to suspect a systematic measurement error among the numerous general practitioners involved, but a tendency towards slightly overestimating length measurements cannot be ruled out. However, a systematic measurement difference between breastfed and formula fed offspring seems unlikely.

The high retention rate generated by the data from the general practitioners virtually excludes selection bias in the material; however selection bias affecting the comparison with the reference populations is still a possibility as the reference standard data have been derived differently.

While the association of breastfeeding and low BMI did have statistical significance, the question of causality cannot be assessed. The estimated associations may stem from reverse causation, i.e. that mothers adjust their feeding regimen according to observed growth. Besides, confounding is always a conspicuous risk in observational cohort studies. We were able to adjust for smoking and maternal pregestational BMI, but no data on socio-economic status were available. However, confounding from this source may primarily be mediated through smoking and obesity. Potential known and unknown confounders including paternal conditions and ethnic differences may interfere with results. To improve the clinical interpretability of the association analysis we chose to translate SD-scores to approximated kg/m^2^ (interpretation of Table 3, results section) even though this comes at a diminutive cost of strict mathematical precision.

The cohort consisting of 131 individuals is small when conducting analyses of associations or sub-groups. However, as a very rough rule of thumb, small studies imply more problems interpreting negative than positive results, and despite the study size, the analyses of association between breastfeeding and BMI reached statistical significance. Additionally, it is reassuring that previous reports of breastfeeding difficulties associated with maternal BMI and following prelabour Caesarean delivery seem to be confirmed. The study contributes to the existing literature but some subgroup analyses are too small for independent conclusions. Particularly, the partially breastfed groups may be quite heterogeneous, and the study has little power to assess optimality of mixtures of breastfeeding and other nutritional sources in the range between full breastfeeding and none.

## Conclusion

At a regional Danish Centre 131 offspring from nulliparous women with GDM managed by life-style interventions were followed-up for 5 months. Breastfeeding rates were lower and complementary feeding introduced earlier than generally reported in Denmark. Lowest breastfeeding rates were displayed following Cesarean delivery prior to labour. In general, the length of the children was longer and the BMI lower compared with standard WHO and Danish childhood references, and breastfeeding was associated with lower BMI. New initiatives to promote breastfeeding and increase breastfeeding rates among Danish women with GDM should be considered.

## Abbreviations

GDM: Gestational diabetes mellitus
BMI: Body mass index
WHO: World Health Organization
